# Efficacy of Antiviral Drugs against Feline Immunodeficiency Virus

**DOI:** 10.3390/vetsci2040456

**Published:** 2015-12-18

**Authors:** Katrin Hartmann, Anita Wooding, Michèle Bergmann

**Affiliations:** Clinic of Small Animal Medicine, LMU Munich, Veterinaerstrasse 13, 80539 Munich, Germany; E-Mails: anitaschwartz@hotmail.com (A.W.); n.bergmann@medizinische-kleintierklinik.de (M.B.)

**Keywords:** FIV, antiviral compounds, treatment, therapy

## Abstract

Feline immunodeficiency virus (FIV) is one of the most common infectious agents affecting cats worldwide .FIV and human immunodeficiency virus (HIV) share many properties: both are lifelong persistent lentiviruses that are similar genetically and morphologically and both viruses propagate in T-lymphocytes, macrophages, and neural cells. Experimentally infected cats have measurable immune suppression, which sometimes progresses to an acquired immunodeficiency syndrome. A transient initial state of infection is followed by a long latent stage with low virus replication and absence of clinical signs. In the terminal stage, both viruses can cause severe immunosuppression. Thus, FIV infection in cats has become an important natural model for studying HIV infection in humans, especially for evaluation of antiviral compounds. Of particular importance for chemotherapeutic studies is the close similarity between the reverse transcriptase (RT) of FIV and HIV, which results in high *in vitro* susceptibility of FIV to many RT-targeted antiviral compounds used in the treatment of HIV-infected patients. Thus, the aim of this article is to provide an up-to-date review of studies on antiviral treatment of FIV, focusing on commercially available compounds for human or animal use.

## 1. Introduction

FIV can induce immunosuppression, which predisposes cats to secondary infections, stomatitis, neuropathies, and tumors. FIV does not directly cause severe clinical disease in most infected cats. Thus, FIV-infected cats can live with a good quality of life for many years with preventive health measures, and overall survival time of infected cats is not necessarily shorter than in uninfected cats [1,2,3]. The most important strategy for managing FIV-infected cats is treatment of secondary infections. In cats with recurrent infections despite aggressive management, additional treatment with antiviral drugs (e.g., plerixafor and/or zidovudine) can be considered. Cats with no identifiable secondary diseases or with secondary disease that has been treated successfully might still suffer from problems likely associated with FIV, such as neurologic abnormalities or stomatitis; in these cases, antiviral treatment (e.g., with zidovudine) is also an option.

Antiviral compounds interfere with certain steps in the viral replication cycle. Based upon steps targeted, the drugs can be assigned to different drug classes [4,5]. This review covers the most common drugs used for treatment of FIV infection (Table A1): reverse transcriptase inhibitors (RTIs), which inhibit the retroviral enzyme reverse transcriptase (RTIs); drugs that inhibit other viral enzymes, such as DNA or RNA polymerases, thereby interfering with virus genome replication or with proteinases necessary for splitting precursor proteins during viral assembly (nucleotide synthesis inhibitors); drugs that target viral entry by binding to specific receptors that the virus uses for adsorption to the target cell, by acting as fusion inhibitors preventing conformational changes of the virus necessary for the fusion process, or by interfering with viral uncoating (receptor homologues/antagonists) [4,6]; drugs that inhibit integration by inhibiting the retroviral enzyme integrase (integrase inhibitors); drugs that inhibit viral replication by inhibiting the retroviral enzyme protease (protease inhibitors); and interferons.

### Reverse Transcriptase Inhibitors

The most commonly used antiretroviral drugs in human and veterinary medicine are RTIs. There are three categories of RTIs: nucleoside analogue RTIs (NARTIs, Section 1), nucleotide analogue RTIs, Section 2, and non-nucleoside RTIs (NNRTIs, Section 3) [5,7]. A nucleoside consists of a nitrogenous base covalently attached to a sugar (ribose in RNA, 2-deoxyribose in DNA), and a nucleotide consists of a nitrogenous base, a sugar, and a phosphate group. Nucleic acid (RNA and DNA) contains a chain of nucleotides covalently linked to form a sugar-phosphate backbone with protruding nitrogenous bases. Prior to linkage of a new nucleotide (or monophosphate) to the nucleic acid, three phosphate groups must be bound to the nucleoside (triphosphate), two of which are removed, releasing energy during elongation of the nucleic acid chain [5].

## 2. Nucleoside Analogue Reverse Transcriptase Inhibitors

NARTIs are the most widely used antiviral compounds in both human and veterinary medicine. NARTIs are molecules that are similar to the “true” nucleosides and require intracellular phosphorylation for activation. Due to their structural similarities, they can bind to the active center of enzymes (e.g., RT, other polymerases) and block enzyme activity. Many of these analogues are also integrated into growing DNA or RNA strands, but because of small differences in the molecular structure, chain termination results or nonfunctional nucleic acids are produced [5,8,9]. NARTIs are accepted as false substrates by viral enzymes as well as by cellular enzymes, which is the main reason for their toxicity [10].

### 2.1. Zidovudine

Zidovudine (3′-azido-2′,3′-dideoxythymidine, AZT) was first synthesized in the 1960s [11] as a potential anticancer drug. In 1985 it was shown to be effective against HIV [12] and became the first drug approved for treatment of HIV infection [13].

The anti-FIV activity of zidovudine has been assessed in numerous *in vitro* studies in different cell systems [14,15,16,17,18,19,20,21,22,23,24,25,26]. The first *in vitro* study was carried out in 1989, when North and coworkers showed that zidovudine inhibited FIV replication in Crandell-Rees feline kidney (CRFK) cells. The susceptibility of FIV to zidovudine was similar to that of HIV [27]. There is evidence that FIV can become resistant to nucleoside analogues, as is the case in HIV. Zidovudine-resistant FIV mutants can arise after only six months of use, and a single-point mutation in the FIV gene is responsible for resistance [10].

*In vivo*, zidovudine can reduce plasma viral load, improve the immunologic and clinical status of FIV-infected cats, increase quality of life, and prolong life expectancy [16]. In placebo-controlled trials, zidovudine improved stomatitis and increased the CD4/CD8 ratio in naturally FIV-infected cats. In some cats with FIV-associated neurologic signs, marked improvement was reported within the first days of therapy [28,29].

Zidovudine not only inhibits RT, but also cellular polymerases, and this can lead to bone marrow suppression. Regular blood cell counts are necessary during zidovudine treatment because non-regenerative anemia is a common side effect [28]. Cats with bone marrow suppression should not be treated with zidovudine. Most FIV-infected cats treated with zidovudine for as long as two years tolerated the drug well. The hematocrit can decline within three weeks of initiating treatment to approximately 50% of baseline but increases afterwards in most cases, even without discontinuation of treatment. If the hematocrit drops below 20%, discontinuation of treatment is recommended, and anemia usually resolves within a few days. Other side effects in cats, including vomiting or anorexia, are rare [28].

### 2.2. Stavudine

Stavudine (2′,3′-didehydro-2′,3′-dideoxythymidine, d4T) is another drug effective against HIV. It was approved for treatment of HIV infection in 1994, but in recent years has been replaced in most multi-drug treatment protocols by compounds with fewer side effects [30,31,32,33,34].

Stavudine is active against FIV *in vitro* [18,19,20,23,26,35,36]. Mutants of FIV that are resistant to stavudine and cross-resistant to several other antivirals, including zidovudine, have been detected. Resistance is caused by a single-point mutation in the RT-encoding region of the *pol* gene [26]. No *in vivo* data in FIV-infected cats have been published.

### 2.3. Didanosine

Didanosine (2′,3′-dideoxyinosine, ddl) was shown to be active against HIV in 1986 [37]. In the United States, it was the second drug to be approved for treatment of HIV and has been on the market since 1991 [5]. 

Didanosine is active against FIV *in vitro* [14,18,20,21,22,23,24,26,38]. In one experimental *in vivo* study, FIV replication was significantly suppressed in animals treated with didanosine, but treatment contributed to the development of antiretroviral toxic neuropathy [39].

### 2.4. Lamivudine

Lamivudine (2R,cis-4-amino-l-(2-hydroxymethyl-1,3-oxathiolan-5-yl)-(1H)-pyrimidin-2-one, 3TC) is also an anti-HIV drug, approved in 1995 [40]. 

Lamivudine is active against FIV *in vitro* [3,20,21,23,38,41]. A combination of zidovudine and lamivudine had synergistic anti-FIV activities in cell cultures [41]. FIV mutants resistant to lamivudine and containing a point mutation in the RT gene were selected *in vitro* and showed cross resistance to zidovudine [23]. 

In one *in vivo* study, experimentally FIV-infected cats were treated with a high-dose zidovudine/lamivudine combination, which protected some cats from infection when treatment was started before virus inoculation. However, zidovudine/lamivudine treatment showed no anti-FIV activity in chronically infected cats. Severe side effects, including fever, anorexia, and marked hematologic changes, were observed in some of the cats with this high-dose dual-drug treatment [41]. Thus, high-dose lamivudine treatment alone, or in combination with zidovudine, is not recommended in naturally FIV-infected cats.

### 2.5. Emtricitabine

Emtricitabine (2',3'-deoxy-5-fluoro-3'-thiacytidine, FTC) is structurally similar to lamivudine and was licensed by the FDA in 2003 [40]. *In vitro*, antiviral efficacy has been demonstrated against FIV [17,20,21,22], but to date there have been no *in vivo* studies in FIV-infected cats.

### 2.6. Abacavir

Abacavir ((1*S*,4*R*)-4-[2-amino-6-(cyclopropylamino)-9H-purin-9-yl]-2-cyclopentene-1-methanol, ABC) was shown to be active against HIV in 1986 and belongs to the FDA-approved anti-HIV compounds [40]. Abacavir is active against FIV *in vitro,* but had higher levels of cytotoxicity than other compounds, such as didanosine and amdoxovir [16,20]. There are no *in vivo* studies of this drug in FIV-infected cats.

## 3. Nucleotide Analogue Reverse Transcriptase Inhibitors

Similar to NARTIs, NtARTIs also interact with the catalytic site of RT and are incorporated into the elongating proviral DNA strand, causing chain termination [5,42]. They compete with natural nucleotides and therefore function as competitive substrate inhibitors. However, in contrast to NARTIs, NtARTIs already contain one phosphate group and thus need only two intracellular phosphorylation steps for conversion to their active forms because the first and often rate-limiting phosphorylation step is unnecessary [5,42,43].

### 3.1. Adefovir

Adefovir (2-(6-amino-9H-purin-9-yl)-ethoxy-methyl-phosphonic acid, PMEA) is active against herpesviruses, hepadnaviruses (hepatitis B), and retroviruses [44]. Adefovir is not licensed as an HIV drug, but is currently available as an oral formulation (bis-POM PMEA) approved for the treatment of chronic hepatitis B. Adefovir belongs to the acyclic nucleoside phosphonates, in which the alkyl side chain of purines and pyrimidines is linked to a modified phosphate moiety and a C-P phosphonate linkage replaces the normal O5′-P phosphate linkage [43,45]. This phosphonate bond is not hydrolysable, which makes it more difficult to cleave off these compounds once they have been incorporated at the 3′-terminal end of the elongating proviral DNA strand [5]. Adefovir inhibits FIV replication *in vitro* [46]. 

Several studies have investigated the efficacy of adefovir in either experimentally and naturally FIV-infected cats [47,48,49,50,51,52,53]. A few of those studies showed some efficacy, but also reported severe side effects, mainly non-regenerative anemia. In a recent study, adefovir was administered to FIV-infected cats in a six-week placebo-controlled, double-blinded clinical trial; ten cats received adefovir (10 mg/kg SC twice weekly) and ten cats received placebo. There was no decrease in proviral or viral loads in treated cats, and treated cats developed a progressive, sometimes life-threatening anemia, which is a common adverse effect of NtARTIs [53]. This shows that results obtained in experimental studies cannot always be applied to a field situation and emphasizes the importance of controlled clinical field trials. Based on the lack of efficacy in the recent placebo-controlled field trial and the side effects, adefovir cannot be recommended for treatment of FIV-infected cats.

### 3.2. Tenofovir

Currently, the only approved NtARTi for the treatment of HIV infection is tenofovir disoproxil fumarate (TDF), the prodrug of tenofovir ((R)-9-(2-phosphonylmethoxypropyl)adenine, (R)-PMPA), which is also a member of the acyclic nucleoside phosphonates [43,45]. The antiviral spectrum of tenofovir (2R-1-(6-amino-9H-purin-9-yl)-propan-2-yl-oxy-methyl-phosphonic acid, PMPA) is narrower than that of adefovir; it does not encompass herpesviruses, but is confined to hepadna- and retroviruses [44]. Tenofovir disoproxil fumarate has become one of the most commonly used drugs in HIV therapy since its licensing in 2001 [5,9].

Tenofovir is effective against FIV *in vitro* [25,45], and there is some evidence that tenovovir might have greater anti-FIV efficacy with less cytotoxicity than other antiretroviral compounds, including adefovir [45,54]. However, *in vivo* studies are lacking and should be a focus of future research.

## 4. Non-Nucleoside Reverse Transcriptase Inhibitors

Most of the NNRTIs are highly specific for HIV-1 and are not active against other retroviruses, including HIV-2 and FIV [7,42]. Unlike NARTIs and NtARTIs, which bind to the catalytic site of RT, non-nucleoside RT inhibitors interact with an allosteric site of the enzyme [5] and are not incorporated into the proviral DNA strand [42]. They are classified as non-competitive inhibitors of RT and do not require intracellular activation for inhibition of the enzyme [8,42]. NNRTIs are a group of structurally diverse compounds that all bind a single site of the RT [55]. The interaction with the allosteric site which is located in close proximity to the catalytic site, leads to a number of conformational changes of the RT [55,56]. Among other effects, these changes cause a reduction in the interaction between the DNA primer and the polymerase domain of the enzyme and thus, inhibit virus replication [55,56].

Three of the FDA-approved NNRTIs (nevirapine, delavirdine, efavirenz) have been shown not to be effective against FIV *in vitro* [40,57]. *In vivo* studies have not been performed, presumably because of the lack of *in vitro* efficacy [7,57]. Only one old NNRTI, suramin, with a broad antiviral spectrum has been used in veterinary medicine. A major breakthrough in the treatment of FIV would be the discovery of more NNRTIs with activity against FIV RT.

### Suramin

Suramin (1-(3-benzamido-4-methylbenzamido)-naphthalene 4,6,8-trisulfonic acid sym-3′-urea sodium salt), a sulfated naphthylamine and trypan red derivative, is one of the oldest known antimicrobial agents. It has been used as an antitrypanosomal agent and for the treatment of some tumors, such as prostate cancer [58]. It also has an inhibitory effect on the RT activity of retroviruses and has also been used in humans with HIV infection [59]. Suramin inhibits RT by interacting with the template-primer binding site of the enzyme. Thus, it competitively binds to the primer binding site (without being a nucleoside analogue) and inhibits the template-primer binding that is necessary for DNA elongation. Suramin can therefore be classified as an NNRTI [60].

Suramin is effective against feline leukemia virus (FeLV) *in vivo* [61,62], and thus, could potentially be active against FIV, although this has not been investigated.

Suramin is associated with a significant number of severe side effects in humans, such as nausea and anaphylactic shock as immediate reactions during administration and peripheral neuritis leading to palmar-plantar hyperesthesia, photophobia, skin reactions, agranulocytosis, hemolytic anemia, and destruction of the adrenal cortex as later side effects [58,59,63,64,65]. In cats with FeLV infection, the major adverse effects of suramin were transient vomiting and anorexia [61].

## 5. Nucleotide Synthesis Inhibitors

Nucleotide synthesis inhibitors prevent synthesis of nucleotides through various mechanisms. They have a broad spectrum of activity but are associated with marked toxicity mainly because they are non-selective and therefore also interfere with normal cellular nucleotide synthesis. Some, for instance foscarnet, interfere with the exchange of pyrophosphate from deoxynucleoside triphosphate during viral replication by binding to RT or DNA polymerase, thereby preventing nucleotide synthesis [66]. Others, such as ribavirin, inhibit inosine monophosphate dehydrogenase after intracellular phosphorylation, which in turn leads to inhibition of guanosine monophosphate.

### 5.1. Foscarnet

Foscarnet (phosphonoformic acid, PFA) has broad-spectrum antiviral activity against DNA and RNA viruses, including retroviruses. It is FDA-approved for the treatment of HIV-associated cytomegalo and herpes simplex virus infections in humans [67]. Foscarnet is usually administered intravenously by continuous intravenous infusion because of its short half-life, which has also been demonstrated in cats [68]. Oral administration of the drug is possible but can result in irritation of mucous membranes and oral bleeding. Foscarnet has many side effects, including nephrotoxicity and myelosuppression, in both humans and cats. It also is toxic to epithelial cells and mucous membranes, resulting in gastrointestinal side effects and genital epithelium ulceration. In addition, it chelates various cations, which can lead to hypocalcemia, hypomagnesemia, and hypokalemia [69,70].

*In vitro*, foscarnet has been shown to be active against FIV, but foscarnet-resistant FIV strains can develop [14]. No *in vivo* studies in FIV-infected cats have been carried out, likely because of the severe side effects and necessity for continuous intravenous administration of the drug. 

### 5.2. Ribavirin

Ribavirin (1-β-d-ribofuranosyl-1 H-1,2,4-triazole-3-carboxamide, RTCA) has marked *in vitro* antiviral activity against a variety of DNA and RNA viruses [71]. Systemic administration of ribavirin is limited in cats because of side effects [72]. Sequestration of ribavirin within erythrocytes results in hemolysis, even when low doses of the drug are used [73,74]. In addition, there is a dose-related toxic effect on bone marrow, primarily on megakaryocytes, resulting in thrombocytopenia and hemorrhage. With prolonged ribavirin treatment or at higher doses, the production of erythrocytes and neutrophils also is suppressed. Ribavirin also can induce hepatic toxicity. An attempt to decrease the toxicity of ribavirin by incorporating it into lecithin-containing liposomes and administering it at lower doses was not successful [75]. 

Ribavirin is active against many viruses *in vitro*, including FIV [23,76]. Therapeutic concentrations are difficult to achieve *in vivo* because of toxicity [74]. To date, the efficacy of ribavirin has not been investigated in FIV-infected cats.

## 6. Receptor Homologues/Antagonists

Receptor homologues/antagonists bind to the virus or to the cellular receptor, leading to inhibition of viral cell-surface binding. Most of the receptor homologues/antagonists are highly selective for HIV and not useful in veterinary medicine. An exception is the class of antiviral compounds called bicyclams, which have been used in cats with FIV infection. Bicyclams act as potent and selective CXC chemokine receptor 4 (CXCR4) antagonists [77,78]. Chemokine receptors belong to the group of seven transmembrane-proteins that enable signal transmission through rapid influx of calcium into the cell. They are essential co-receptors for HIV as well as for FIV during infection of CD4+ lymphocytes [79,80]. By binding to CXCR4, bicyclams prevent interaction of CXCR4 with other ligands, thereby inhibiting the entry of HIV or FIV into the cell [81,82,83].

### Plerixafor

Plerixafor (1,1′-(1,4-phenylenbismethylene)-bis(1,4,8,11-tetraazacyclotetradecane)-octachlo-ride dehydrate, AMD3100) is the bicyclam prototype compound. It is not marketed as an anti-HIV drug, but is used in humans for stem cell mobilization [84]. 

Plerixafor is active against FIV *in vitro* [82]. In a placebo-controlled double-blinded clinical trial, treatment of naturally FIV-infected cats with plerixafor resulted in a significant decrease in proviral load in treated cats when compared to the placebo group. There was a concomitant decrease in serum magnesium levels, which did not produce any clinical consequences. Development of resistance of FIV isolates to plerixafor did not occur during treatment [53]. In cats, plerixafor is administered at a dosage of 0.5 mg/kg every 12 h. Monitoring of magnesium and calcium levels should be performed at regular intervals during treatment [53]. Further studies investigating the potential of this promising drug are needed.

## 7. Protease Inhibitors

Protease inhibitors (PI) specifically bind to the active site of the protease and therefore prevent viral replication. Several PIs have been used for successful treatment of HIV. Nevertheless, side effects and development of viral resistance were found during treatment, and therefore additional compounds that bind to sites other than the active site of the protease have been developed [85,86,87].

### 7.1. Tipranavir

Tipranavir (*N*-[3-[(1*R*)-1-[(2*R*)-6-hydroxy-4-oxo-2-(2-phenylethyl)-2-propyl-3*H*-pyran-5-yl]propy l]phenyl]-5-(trifluoromethyl)pyridin-2-sulfonamid) was approved in 2005, and is used as an anti-HIV compound. The drug was shown to be active against FIV *in vitro*.Tipranavir completely prevented FIV replication [85,86]. No studies in FIV-infected cats exist so far, and further studies are needed to investigate the potential of tipranavir in naturally infected cats.

### 7.2. Lopinavir

Lopinavir (2*S*)-*N*-[(2*S*,4*S*,5*S*)-5-[2-(2,6-dimethylphenoxy)acetamido]-4-hydroxy-1,6-diphenylhex-an-2-yl]-3-methyl-2-(2-oxo-1,3-diazinan-1-yl)butanamide) is an anti-HIV compound, approved in 2001. The drug was shown to be active against FIV *in vitro* but did not prevent FIV replication completely [85]. There are no *in vivo* studies in FIV-infected cats.

### 7.3. Atazanavir

Atazanavir (methyl N-[(2R)-1-[2-[(2S,3S)-2-hydroxy-3-[[(2R)-2-(methoxycarbonylamino)-3,3-di-methylbutanoyl]amino]-4-(2,3,4,5,6-pentadeuteriophenyl)butyl]-2-[(4-pyridin-2-ylphenyl)methyl]hy-drazinyl]-3,3-dimethyl-1-oxobutan-2-yl]carbamate) was licensed by the FDA in 2003 and is also used as an anti-HIV compound. Similar to lopinavir, some efficacy of atazanavir was shown against FIV *in vitro*, but there are no *in vivo* studies published so far [85].

## 8. Integrase Inhibitors

The enzyme integrase catalyzes strand transfer (3′-end joining), which inserts both viral DNA ends into a host cell chromosome during proviral DNA integration [5,88]. Once integrated, the provirus persists in the host cell genome and functions as a template for replication of the viral genome, leading to the formation of new viruses [89]. The high degree of conservation of integrase-active sites across many retroviruses suggests that FIV might also be sensitive to integrase inhibitors [90]. Integrase inhibitors act through inhibition of integration of the proviral DNA that is produced by reverse transcription of the viral RNA genome [91].

### Raltegravir

Raltegravir is used as an anti-HIV compound. The drug was shown to be active against FIV *in vitro* [92], but FIV was less susceptible to raltegravir than HIV [92]. 

No studies in FIV-infected cats exist so far. Although there are no *in vivo* studies on the efficacy of raltegravir in FIV-infected cats, the drug recently was shown to be effective against FeLV and was safe in cats [93].

## 9. Interferons

Interferons (IFNs) are polypeptide molecules with various biological functions [94]. They play an important role in mediating antiviral and antigrowth responses and in immune response modulation [95]. They can be divided into type I and type II IFNs, both of which have antiviral properties. Type I IFNs, including IFN-α, IFN-β, and IFN-ω, are produced by virus-infected cells [94,96], whereas type II IFN, consisting of only IFN-γ, is produced by activated T lymphocytes and natural killer cells in response to recognition of virus-infected cells [97]. IFNs act in an autocrine or paracrine fashion [98] inducing an anti-viral state in non-infected cells. IFNs bind to specific cell surface receptors and result in the transcription of IFN-stimulated genes. The products of these genes are proteins with potent anti-viral properties that interfere with various stages of viral replication [98]. Several studies suggest that retroviral protein synthesis is not affected by IFNs and therefore conclude that the antiviral activity of IFNs is mainly related to interference with later stages of the viral replication cycle such as virion assembly and release [94,99]. Interferons also trigger virus-infected cells to undergo apoptosis by activating gene expression for apoptosis [97,99], which prevents the spread of virus from infected cells and aids in the clearance of virus infection [97]. Human IFNs have been manufactured by recombinant DNA technology and are available commercially. Recombinant feline IFN-ω is on the market in Japan, Australia, and many European countries and is licensed for use in cats and dogs.

### 9.1. Human Interferon-α

Recombinant human interferon-α (rHuIFN-α) has antiviral and immune-modulatory activity. IFN-α is active against many DNA and RNA viruses [98]. There are two common treatment regimens for use of rHuIFN-α in cats: SC injection (10^4^ to 10^6^ U/kg every 24 h) or oral application (1 to 50 U/kg every 24 h). 

Human IFN-α becomes ineffective after three to seven weeks of parenteral use in cats because of the production of neutralizing antibodies [100]. Anti-IFN-α antibody production does not occur with oral administration of IFN-α and therefore this route allows for a longer period of treatment. IFN-α is inactivated by gastric acid and destroyed by trypsin and other proteolytic enzymes in the duodenum [101], which means that direct antiviral effects are unlikely after oral application. However, oral IFN-α appears to have immuno-modulatory activity, because it can stimulate local lymphoid tissue. The release of cytokines by lymphatic cells in the oropharyngeal area triggers a cascade of immunologic responses with systemic effects [102,103,104].

RHuIFN-α has been shown to be active against FIV *in vitro* [105]. Although frequently used in the field for treating FIV-infected cats, controlled studies evaluating the effect of parenteral administration of rHuIFN-α in FIV-infected cats have not been conducted. 

Use of oral rHuIFN-α in 24 ill, naturally FIV-infected cats (50 U/kg applied to the oral mucosa daily for seven days on alternating weeks for six months, followed by a two-month break, and then repetition of the six-month treatment) resulted in improvement of clinical signs (e.g., fever, lymphadenopathy, opportunistic infections) in a placebo-controlled, double-blinded study [106]. However, proviral and viral loads were not monitored during thiat study and therefore it is impossible to conclude whether treatment with rHuIFN- α had indeed an effect on FIV, or rather on secondary infections.

### 9.2. Feline Interferon-ω

Recombinant feline interferon-ω (rFeIFN-ω), the corresponding feline interferon, is licensed for use in veterinary medicine in Japan, Australia, and some European countries. It can be used in cats for long periods without antibody development, and no major severe side effects have been reported [107].

IFN-ω inhibits FIV replication *in vitro* [105]. One placebo-controlled, multicenter study that investigated the effect of parenteral rFeIFN-ω against FIV infection in 62 naturally FIV-infected cats (treated with 10^6^ U/kg SC q 24 h on five consecutive days) did not find a difference in the survival rate in treated cats. However, some improvement in clinical scores, including eight categories of clinical signs (rectal temperature, behavior, appetite, thirst, dehydration, mucous membrane appearance, stomatitis, and death) as well as improvement in laboratory abnormalities (leukopenia, leukocytosis, and anemia) occured [107]. In another study, which evaluated naturally FIV-infected cats housed in a shelter, some clinical improvement was observed after parenteral rFeIFN-ω (10^6^ U/kg SC q 24 h on FIVe consecutive days for three cycles), but this study lacked a placebo control. In that same study, hematologic values remained within reference intervals, and there were no biochemical abnormalities associated with rFeIFN-ω treatment [96]. 

A recent study evaluated the use of oral administration of rFeIFN-ω for the treatment of eleven client-owned, naturally FIV-infected cats with clinical signs [108]. The treatment protocol was 10^5^ U/cat PO q 24 h for 90 consecutive days, administered by the cats’ owners. A historical retrospective group was used as a control for comparison (10^6^ U/kg SC q 24 h on five consecutive days for three cycles), but a placebo group was not included. Treatment with oral rFeIFN-ω resulted in a significant improvement in clinical scores (e.g., oral lesions, coat appearance, body condition score, and ocular discharge) after treatment. In addition, there was no significant difference between the SC historical control group and the PO group, suggesting that oral administration of rFeIFN-ω might be a viable and less expensive alternative [109]. In a recently published study that assessed viremia, provirus load, and blood cytokine profile in naturally FIV-infected cats treated with oral rFeIFN-ω (10^5^ U/cat PO q 24 h for 90 days) or with subcutaneous rFeIFN-ω (10^6^ U/cat SC q 24 h for 5 consecutive days in three courses), no change in the level of viremia or in most cytokine levels was found; a placebo control group was not included [109]. The fact that virus load remained unchanged but some clinical improvement was observed in earlier studies suggests that rFeIFN-ω has an effect on secondary infections rather than on FIV itself [94]. As there are major differences in outcomes of the different studies on feline IFN-ω in FIV-infected cats. Thus, a definitive conclusion cannot be drawn without additional randomized, placebo-controlled, and double-blinded studies that include a sufficiently high number of naturally FIV-infected cats.

## 10. Conclusions

Unfortunately, the efficacy of antiviral compounds for the treatment of FIV in cats has been generally poor. The duration of treatment in many clinical trials was relatively short and might have been inadequate for infections with a long clinical course. In addition, it is difficult to compare treatment results of cats infected experimentally and kept under laboratory conditions and pet cats infected with field strains of FIV. Therefore, further well-designed double-blinded, placebo-controlled trials using antiviral drugs in naturally FIV-infected cats are needed to determine the efficacy and side effects of different antiviral compounds.

## Appendix

**Table A1:** Treatment options (antiviral drugs) for FIV-infected cats (including EBM grades for judgment of the available efficacy data; EBM grades used according to the European Advisory Board of Cat Diseases (ABCD).

Drug	Efficacy *In Vitro*	Efficacy *in Vivo*	Author’s Personal Opinion	Ebm Level (I–Iv)
***Nucleoside Analogue—Reverse Transcriptase Inhibitors***				
Zidovudine (AZT)	yes [27,14,16,17,18,19,20,22,23,24,25,26,38]	yes [76,28,29]	effective in some cats (e.g., with stomatitis, neurological disorders)	I
Stavudine (d4T)	yes [14,18,20,21,22,23,26,76]	nd	possibly effective, but no data in cats available	IV
Didanosine (ddI)	yes [14,18,19,20,21,22,23,24,26,38]	yes [39]	effective in one experimental study, but neurologic side effects	II
Zalcitabine (ddC)	yes [14,15,17,19,21,22,23,26,47]	nd	possibly effective, but toxic	IV
Lamivudine (3TC)	yes [16,17,20,21,22,23,76]	no [41]	not very effective, toxic in high dosages	II
Emtricitabine (FTC)	yes [17,20,21,22]	nd	possibly effective, but no data in cats available	IV
Abacavir (ABC)	yes [20]	nd	possibly effective but toxic	IV
***Nucleotide Analogue Reverse—Transcriptase Inhibitors***				
Adefovir (PMEA)	yes [28]	no [47,48,49,50,51,52]	effective in some cats, but relatively toxic	I
Tenofovir (PMPA)	yes [25,35,45]	nd	possibly effective, but also likely relatively toxic	IV
***Non-Nucleoside Reverse Transcriptase Inhibitor***				
Suramin	no	nd	likely too toxic	IV
***Nucleotide Synthesis Inhibitors***				
Foscarnet (PFA)	yes [14]	nd	effective *in vitro*, but too toxic	IV
Ribavirin	yes [23,76]	nd	possibly effective, but too toxic in cats	IV
***Receptor Homologues/Antagonists***				
Plerixafor	yes [82]	yes [53]	some effect in a study in privately-owened cats (thus, can be considered as treatment)	I
***Protease Inhibitors***				
Tipranavir	yes [85]	nd	potentially effective, but no *in vivo* data available	IV
Lopinavir	yes [85]	nd	likely ineffective	IV
Atazanavir	yes [85]	nd	likely ineffective	IV
***Integrase Inhibitors***				
Raltegravir	yes [85]	nd	possibly effective, but no data in FIV-infected cats available	IV
***Interferons***				
Human interferon-α (IFN-α) SC high dose (10^6^ U/kg q 24 h on five consecutive days)	yes [105]	no [100]	likely ineffective	IV
SC intermediate dose (10^5^ U/kg q 24 h for 90 days)	yes [105]	nd	likely ineffective	IV
PO low dose (50 U/kg every 24 h for long-term period)	yes [105]	yes [106]	some efficacy (most likely through effect on secondary infection)	I
Feline interferon-ω (IFN-ω) SC high dose 10^6^ U/kg q 24 h on FIVe consecutive days	yes [105]	yes [107]	some improvement of clinical signs (most likely through effect on secondary infection)	I
PO intermediate dose 10^5^ U/cat q 24 h for 90 consecutive days	yes [105]	yes [108]	potentially some efficacy (most likely through effect on secondary infection)	III
PO low dose 10^5^ U/cat q 24 h for 90 consecutive days	yes [105]	nd	potentially effective (most likely through effect on secondary infection)	IV

*FIV*, feline immunodeficiency virus; *nd*, not determined. **EBM, evidence based medicine [4,110]:** EBM grade I = This is the best evidence, comprising data obtained from properly designed, randomized controlled clinical trials in the target species (in this context cats). EBM grade II = Data obtained from properly designed, randomized controlled studies in the target species with spontaneous disease in an experimental setting. EBM grade III = Data based on non-randomized clinical trials, multiple case series, other experimental studies, and dramatic results from uncontrolled studies. EBM grade IV = Expert opinion, case reports, studies in other species, pathophysiological justification.
